# Real-time locomotion mode detection in individuals with transfemoral amputation and osseointegration

**DOI:** 10.1186/s12984-025-01672-2

**Published:** 2025-06-24

**Authors:** Bahareh Ahkami, Morten B. Kristoffersen, Max Ortiz-Catalan

**Affiliations:** 1 Center for Bionics and Pain Research, Mölndal, Sweden; 2 Department of Electrical Engineering, Chalmers University of Technology, Gothenburg, Sweden; 3 Department of Orthopaedics, Institute of Clinical Sciences, Sahlgrenska Academy, University of Gothenburg, Gothenburg, Sweden; 4 Prometei Pain Rehabilitation Center, Vinnytsia, Ukraine; 5 Center for Complex Endoprosthetics, Osseointegration, and Bionics, Kyiv, Ukraine

**Keywords:** Electromyography, Myoelectric pattern recognition, Lower limb prostheses, Prosthetics, Osseointegration

## Abstract

**Background:**

Despite notable advancements in prosthetic leg technology, commercially available devices with embedded algorithms utilizing bioelectric signals for prosthetic leg control are lacking. This untapped potential could enhance current prosthetic leg capabilities, enabling more natural movements. However, individuals with short residual limbs have limited available muscle and it has not been investigated if different locomotion modes can be predicted in real-time in this population. Here, we explored the feasibility of using electromyographic signals in individuals with short residual limbs and osseointegrated implants to infer locomotion modes.

**Methods:**

We recorded data from five participants with transfemoral amputation and osseointegration while walking on level ground, stairs, and ramps. Electromyography, acceleration, angular velocity, and ground reaction force were collected using wireless sensors. Two sessions of recordings for offline and real-time evaluation were conducted, with 30 rounds and 15 rounds, respectively. Decoding was performed using a mode-specific, phase-dependent classifier. The method was implemented in LocoD, an existing open-source platform, allowing for further development by the community and allowing easy comparison between different classification algorithms. The evaluation of the platform and prediction algorithm relies on quantifying the transition error, signifying instances where the algorithm falls short in predicting shifts between different walking surfaces.

**Results:**

In this study, a participant exhibited an average error as low as 1.2%, indicating precise predictions. Conversely, the highest average error was found at 23% in a different participant. This variation could be the result of factors related to the amputation such as residual limb length, remaining muscles, and the surgical technique used while performing the amputation, as well as differences in performing the movements. On average, offline classification resulted in a mean error of 5.7%, while the corresponding mean error during online (real-time) evaluation was 11.6%.

**Conclusion:**

Our findings suggest that myoelectric signals can be potentially used in the control of prosthetic legs for individuals with short residual limbs with osseointegrated implants. Further research into understanding and compensating for variations in the locomotion detection accuracy for different participants is crucial.

**Supplementary Information:**

The online version contains supplementary material available at 10.1186/s12984-025-01672-2.

## Background

Lower limb amputation often results in limited mobility and loss of social engagement. With an expected two million people with amputation in the US by 2050, research in prosthetics appears crucial to address this challenge [1]. Over the past few decades, powered lower limb prosthetics have advanced considerably, particularly in hardware development and control algorithms, aiming to enhance user freedom, comfort, and independence [2]. Despite these promising advancements, commercially available powered prosthetic legs today primarily rely on mechanical sensors such as Inertial Measurement Units (IMUs), pressure sensors, and loadcells for control. Devices like the Power Knee (Össur, Iceland) use embedded inertial and load sensors with rule-based algorithms to detect terrain changes and adjust control modes accordingly. Research platforms such as the Open-Source Leg (OSL) [3] have explored more advanced detection strategies, but still depend largely on mechanical sensing. These systems monitor limb orientation, axial loading, and joint angles, but they cannot directly reflect the user’s intention. Critically, bioelectric signals such as electromyography (EMG) are not incorporated into commercial systems yet, despite their potential to enhance user-driven control. EMG signals could overcome many of the limitations associated with mechanical sensors and have shown promise for more intuitive prosthesis control [4, 5]. However, the integration of EMG into lower limb prosthetics remains limited, primarily due to challenges in obtaining reliable signal quality. These challenges include relative motion between the residual limb and the socket, pistoning effects, sweat, electrode displacement, and variability in skin impedance [6, 7].

One critical requirement for improving the usability and safety of EMG controlled powered lower limb prosthetics is the ability to detect locomotion transitions in real time. Inaccurate or insufficient intention detection often results in delayed or wrong mode switching, particularly during transitions such as stair ascent or ramp navigation. Such delays can compromise balance, increase fall risk, and reduce user trust in the prosthesis. Therefore, achieving accurate, real-time classification of user intent is essential to enable timely and seamless adaptation of prosthetic control modes to changing environments. Real-time locomotion mode classification has been successfully demonstrated in people with transfemoral amputation in several studies. Huang et al.. developed real-time intent recognition systems that combined electromyographic (EMG) and mechanical signals for controlling powered transfemoral prostheses during transitions between level walking, stair ascent/descent, and ramp navigation [8, 9]. Further, Zhang et al. proposed optimized source selection methods to improve classification robustness for people with transfemoral amputation during walking and standing tasks [10]. These studies collectively demonstrate the feasibility of real-time, event-driven control in population with transfemoral amputation. Most of these investigations have focused on users with standard residual limb lengths and socket-based attachments, and whether skeletal attachment via osseointegration can alleviate the challenges of placing electrodes with a socket remains unknown.

Beyond people with transfemoral amputation, offline and real-time locomotion classification has also been explored in individuals with transtibial amputation [11–13] and able-bodied individuals [14–17]. These studies confirmed the potential of EMG and multimodal sensor fusion for accurate locomotion mode detection but did not address the unique anatomical and signal acquisition challenges associated with short residual limbs following transfemoral amputation.

Navigating EMG signal acquisition in individuals with short residual limbs presents distinct challenges arising from limited muscle capacity. Frequently, this condition leaves patients unable to effectively utilize traditional socket-based prosthetics or confront difficulties in doing so. Osseointegration (OI) has emerged as a promising solution to provide secure limb attachment in such cases [18]. Furthermore, the potential of OI can be further amplified by integrating it with implanted electrodes, thereby presenting avenues for enhancing prosthetic control [19, 20]. While implanted electrodes have seen application in lower limb prosthetics in a singular study, their full potential and combination with OI remain largely unexplored [21].

Before advancing to invasive strategies, it is essential to first evaluate the feasibility and performance of non-invasive surface EMG-based control in this clinical population. A thorough assessment of sEMG can yield foundational insights into signal quality, decoding feasibility, and classification approaches in individuals with osseointegrated implants. Previous studies have shown that combining EMG with non-biological sensors such as IMUs improves the accuracy of locomotion mode detection in able-bodied participants [9, 22–28].

In the present study, we aim to extend this understanding by validating real-time locomotion mode detection in individuals with transfemoral amputation and short residual limbs who have undergone osseointegration surgery. Using surface EMG recordings from the residual limb, we implemented and evaluated a real-time, event-driven classification algorithm developed within an open-source framework for EMG-based locomotion detection known as LocoD [29]. By conducting this research, we aim to advance the feasibility of non-invasive EMG-based control in this clinically distinct population, paving the way for future improvements in prosthetic functionality, user independence, and quality of life.

## Methods

### Participants

The inclusion criteria for participants were (1) acquired limb loss at the transfemoral level (2) using an osseointegrated implant for connecting their prosthetic leg with the body and (3) being able to walk without assistance. Before joining the study, a detailed explanation of the experimental protocol was provided to all participants, and they provided their informed consent by signing the consent form. Ethical approval was granted by the Sweden ethics committee (number 20-06479). Demographic information of the study participants is reported in Table 1.

### LocoD software platform

LocoD is the open-source, MATLAB-based software platform developed to facilitate the recording, processing, and classification of surface EMG signals for lower limb prosthetic control. The platform includes graphical user interfaces (GUIs) for each stage of the signal processing pipeline, including:

- **Signal acquisition and labeling**, with real-time visualization and manual mode tagging.
- **Pre-processing**, including notch and bandpass filtering tailored to typical surface EMG frequency bands.
- **Gait event detection** using pressure sensors to identify heel contact and toe-off events.
- **Windowing and labeling**, anchored to gait events, to enable phase-dependent classification.
- **Feature extraction** from EMG, IMU, and GRF signals, including time-domain features (e.g., mean absolute value, waveform length, slope changes).
- **Classifier training and evaluation**, supporting mode-specific, phase-dependent models.

In the present study, LocoD was used for both offline classifier training and real-time locomotion mode detection, directly interfacing with the Trigno Wireless EMG system.

The platform is openly available on GitHub, enabling reproducibility, community-driven development, and benchmarking across research groups [29].

### Measurement

Participants walked at a self-selected pace with their own passive prosthetics that they wear in daily life. Electromyography (EMG) signals, Inertial Measurement Unit (IMU) signals, and Ground Reaction Force (GRF) were concurrently recorded from the amputated side (Trigno Avanti, Delsys, USA).

Surface EMG signals were acquired using Trigno Avanti™ wireless sensors (Delsys Inc., USA). Each sensor consists of dry silver electrodes arranged in a parallel-bar configuration, with an interelectrode distance of 10 mm and individual electrode dimensions of approximately 10 mm in length and 1 mm in width. The sensing elements are embedded in a 27 × 37 × 13 mm housing. Sensors were affixed directly to the skin using the manufacturer’s integrated adhesive system, following skin preparation with alcohol wipes to minimize impedance. Electrode placement followed SENIAM guidelines, targeting eight muscles commonly selected for myoelectric control studies: semitendinosus, biceps femoris (long and short heads), tensor fasciae latae, rectus femoris, vastus lateralis, vastus medialis, and gracilis [6]. Muscle identification was performed by palpation during hip movements and imagined knee flexion. All EMG recording procedures adhered to ISEK recommendations regarding electrode specifications and reporting standards.

IMU data were acquired from the integrated 3-axis accelerometers and 3-axis gyroscopes within the Trigno Avanti™ sensors, providing six degrees of freedom per unit. Three IMUs were positioned at anatomically relevant locations: (1) the residual thigh (above the knee), (2) the prosthetic shank, and (3) on the prosthetic foot. This configuration was selected to capture segmental dynamics critical for locomotion mode classification [30].

Ground reaction force (GRF) signals were measured using a single uniaxial force sensor embedded in a custom-made, thin shoe insole placed centrally beneath the prosthetic heel. This sensor enabled detection of gait events, including heel contact and toe-off, which were used to trigger real-time classification.

All EMG, IMU, and GRF signals were sampled at 2000 Hz with 16-bit resolution. Signals were transmitted wirelessly from the Trigno sensors to the Delsys base station, which was interfaced with the data acquisition computer. The shared acquisition system ensured temporal synchronization of all modalities, supporting integrated offline and real-time analyses.

**Table 1:** Demographic information of participants with transfemoral amputation

	Age (years)	Sex	Ratio of residual limb length to intact limb length (from hip to knee)	Prosthetic Device	Osseointegrated Implant
TF001	28	Male	0.5	C-leg	OPRA
TF002	49	Female	0.5	C-leg	OPRA
TF003	48	Male	0.55	C-leg	OPRA
TF004	73	Male	0.55	C-leg	OPRA
TF005	59	Female	0.65	Genium	OPRA

### Experimental protocol

**Fig. 1:**
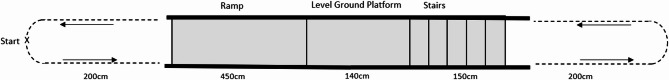
Visual overview of different walking surfaces in the experiment. Participants entered the path by walking, transitioned to an ascending ramp, walked at ground level, descended the stairs, walked, ascended the stairs, transitioned to walking again, and finally descended the ramp

We investigated the decoding of five distinct locomotion. Once equipped with EMG, IMU, and GRF sensors, participants walked a path that included level ground surface, stairs (6 steps, tread 30 cm, riser 10 cm), and a ramp (7° slope). The length of the different walking surfaces is indicated in Fig. 1.

EMG, IMU, and GRF signals were recorded with the Delsys system and transmitted to LocoD [29].

Participants were instructed to initiate walking with their prosthetic limb when transitioning to a new surface to ensure consistency across subjects. For safety, handrails were installed along the ramps and stairs, and participants were permitted to use them if necessary to maintain stability.

Following sensor placement, two recording sessions were conducted on the same day:

### Offline training data collection session

- Participants completed 35 walking rounds along the designated path. This session, lasting approximately 30 to 45 min, was dedicated to collecting training data for classifier development.

### Online evaluation session

- After a 15-minute rest break, during which the LocoD classifier was trained using the collected offline data, participants performed 15 additional walking rounds along the same path. Real-time locomotion mode predictions generated by the trained classifier were displayed on a monitor visible only to the test conductor, who monitored system performance without providing feedback to participants. This session lasted approximately 20 min.

During both sessions, locomotion mode transitions (e.g., walking to ramp ascent) were manually tagged by the test conductor using a keyboard interface. Manual tagging was performed just before the participant entered a new locomotion mode [28, 31]. However, due to inevitable reaction time delays between biomechanical events and manual marker entries, discrepancies could occur between the recorded markers and actual transitions. To mitigate these synchronization issues, an automatic correction process was applied:

For transitions into or out of stair ascent, markers were projected forward to the next toe-off event, and for stair or ramp descent transitions, markers were aligned with the next heel contact. Gait events were extracted from GRF signals using a subject-specific threshold set at 10% of body weight. This alignment ensured accurate labeling of EMG, IMU, and GRF data windows for both offline training and real-time evaluation.

In total, the experimental protocol required approximately 1.5 to 2 h per participant, including preparation activities such as informed consent, protocol explanation, skin cleaning, and sensor placement (approximately 30 min).

### Data processing and decoding

**Fig. 2:**
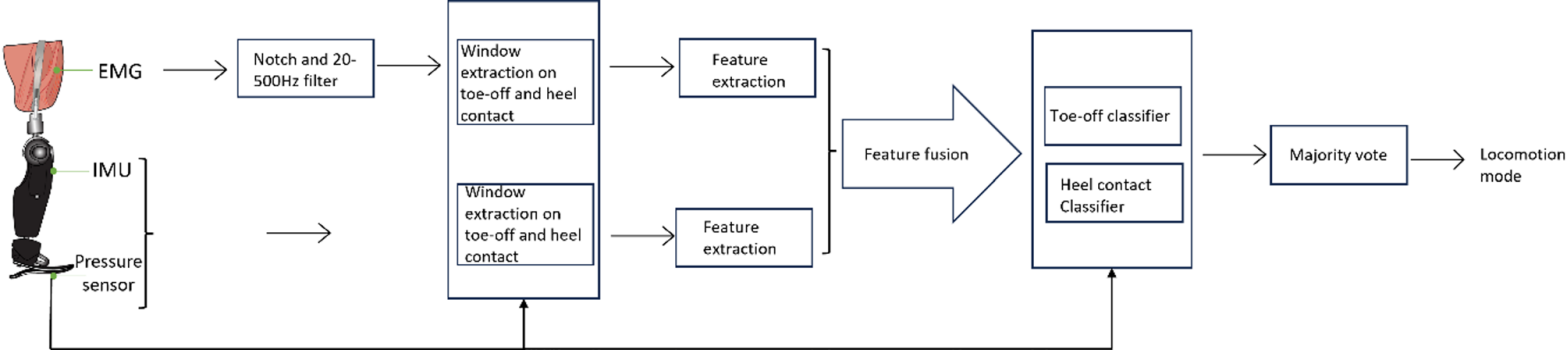
Data processing workflow. Electromyography (EMG), Inertial Measurement Unit (IMU), and pressure sensor data were recorded from the amputated site. The EMG data underwent filtering. Time windows from EMG, IMU, and pressure sensor data were extracted, corresponding to each toe-off and heel contact event. Features were extracted from EMG, IMU, and pressure sensor time windows and merged, forming input for a classifier. Finally, a majority vote was employed to make accurate locomotion detection

To train classifiers to detect different locomotion modes, the recorded data underwent the following steps of processing (Fig. 2):

### Filtering, windowing, and labeling

EMG data were filtered with a 6th order notch filter at 50 Hz to eliminate the power line interference and with a bandpass 4th order Butterworth filter with cut-off frequencies 20–500 Hz [32]. Heel contact and toe-off were identified using a pressure sensor (GRF signal). The threshold for determining toe-off was initially established at 10% of the participant’s body weight. However, recognizing that people with amputation may not distribute their weight across their affected side in the same way as individuals without limb loss, we introduced a precautionary measure. Before recording data, we asked the participants to take a few initial strides and the test conductor checked the signal stream from the pressure sensor. This approach ensured that we could accurately identify key events of heel contact and toe-off. In this study, 10% of the participant’s body weight was a sufficient threshold and there was no need for further adjustments.

Transition markers were automatically aligned with gait events (toe-off or heel contact), as described previously, to ensure accurate labeling for classifier training.

**Fig. 3:**
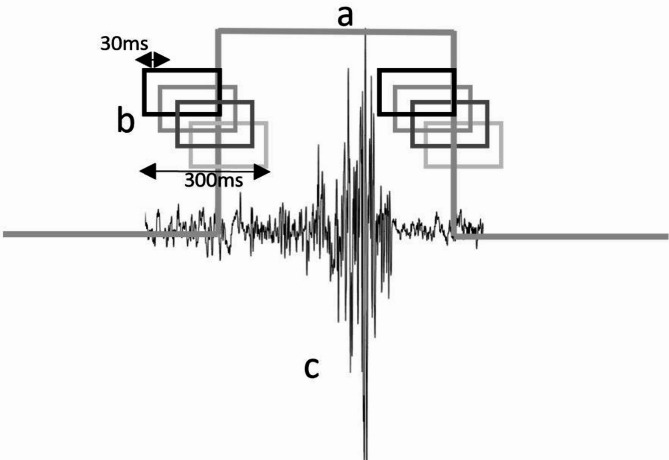
**(a)** Representation of gait events (Toe-off and heel contact) extracted from GRF signal **(b)** 300 milliseconds of extracted data that divided into four 200-millisecond windows with 30-millisecond increments **(c)** EMG signal

Subsequently, a 300-ms segment of data was extracted at each gait event (Heel contact and Toe-off), encompassing 200 ms before and 100 ms after the designated event. Within this 300 ms timeframe, four 200ms windows were created with a 30-ms increment (Fig. 3) [16]. Each window was then labeled based on the realigned markers, indicating its association with a locomotion mode or transition between them.

### Feature extraction

We extracted signal features from the EMG, IMU, and pressure sensor signals to create feature vectors consisting of the waveform length, mean absolute value, zero crossing, and slope change for the EMG signals [16, 33], and mean, maximum, minimum amplitude, and standard deviation for the IMU and pressure sensor data [34, 35]. In total, 108 features were obtained from all modalities for each gait event and combined into a single multimodal feature array.

### Classification architecture

We implemented a mode-specific, phase-dependent classifier architecture in LocoD to infer locomotion modes based on surface EMG, IMU, and GRF signals. Classification was performed at discrete gait events (phase dependent), specifically at heel contact and toe-off, using features extracted from windows surrounding each event. This event-driven structure ensures that predictions are tightly coupled to the gait cycle, improving responsiveness and interpretability.

The classifier followed a state machine design: at each gait event, the previously predicted locomotion mode determined the set of allowable transitions (mode-specific). For level-ground walking, a multi-class classifier was used to allow transitions to ramp ascent, ramp descent, stair ascent, or stair descent. For all other locomotion modes, including ramp ascent, ramp descent, stair ascent, and stair descent, binary classifiers determined whether to remain in the current mode or transition back to level walking. This structure reflects realistic gait progression and disallows biomechanically implausible transitions, such as stair ascent directly to ramp descent [27, 34].

**Fig. 4:**
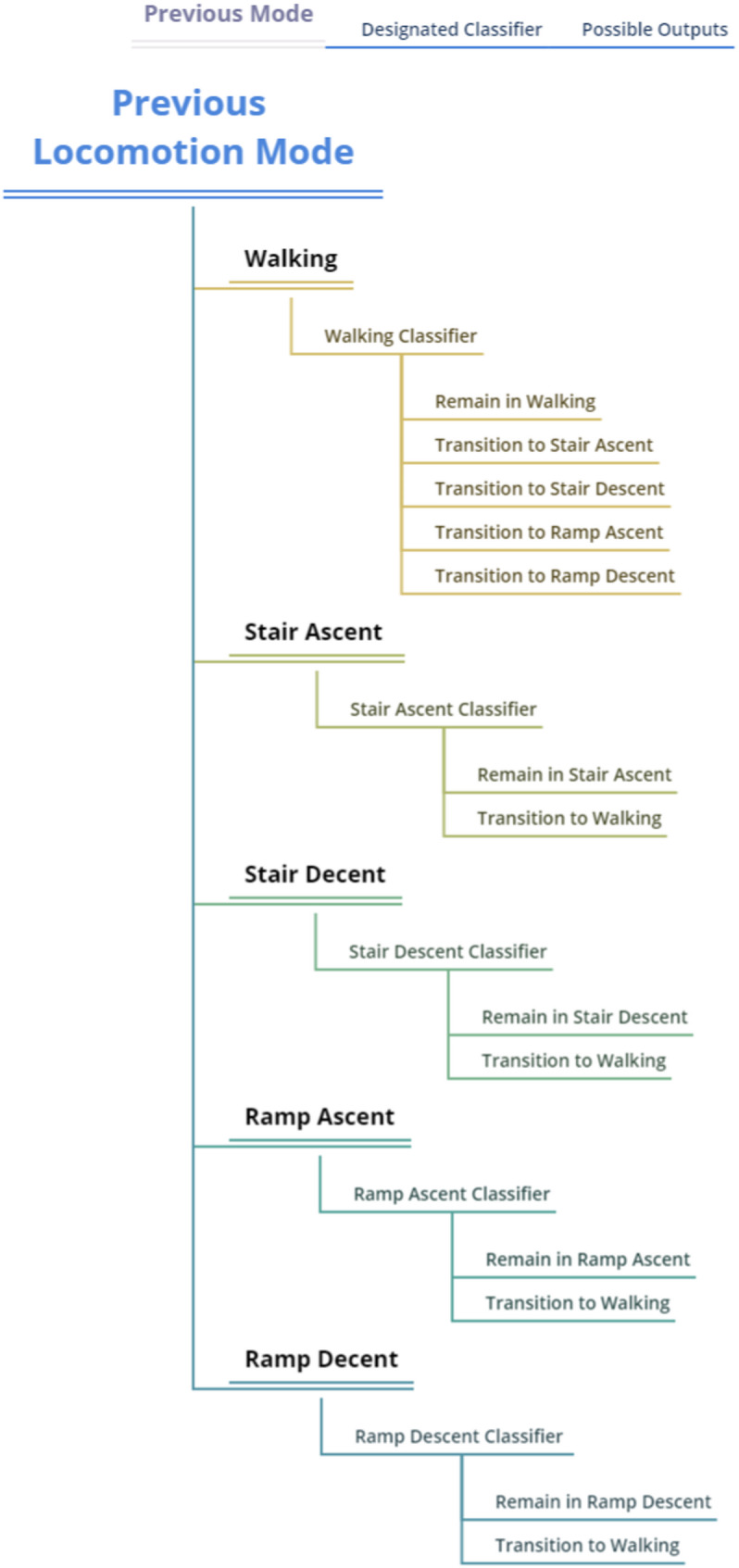
The diagram illustrates the mode-specific, phase-dependent structure of the classifier. At each gait event (toe-off or heel contact), the system evaluates the current locomotion mode and permits transitions only to biomechanically plausible subsequent modes. All transitions route through level-ground walking, which acts as a central hub, preventing direct mode changes between non-adjacent conditions (e.g., stair descent to ramp ascent). This constrained architecture enhances classification stability, reduces misclassification during ambiguous phases, and supports real-time implementation with limited training data

Overall, ten distinct classifiers were trained, corresponding to each combination of locomotion mode (five modes) and gait event (heel contact and toe-off), with separate models for each phase. By constraining transitions to plausible options, the state machine reduced the likelihood of misclassifications. While an unconstrained model allowing free transitions between any mode could theoretically be implemented, it would require a much larger dataset to avoid overfitting. Given the fixed experimental path and limited number of participants, the state-machine approach provided an effective balance between flexibility and robustness. Figure 4 illustrates the allowable transitions between locomotion modes as implemented in the classifier logic.

### Pattern classification algorithm

The classifier used for each mode was based on Linear Discriminant Analysis (LDA). 80% of the features were used randomly to train the classifier and the remaining 20% was used to test the classifier. The trained models were then used to predict the recorded windows in real time. During the training phase of the classifier for real-time predictions, we did not employ a cross-validation method.

For reporting offline classification, a 10-fold cross-validation was performed independently for each participant on the data collected during the offline session. This approach ensured a balanced partitioning of training and testing data across all locomotion modes. We did not perform cross-subject generalization (e.g., leave-one-subject-out validation), as the study’s primary goal was to assess the feasibility of individualized classifiers in real-time conditions, consistent with prior early-stage lower-limb prosthesis research.

The real-time evaluation employed the classifiers trained on each participant’s offline session data to predict locomotion modes during the subsequent online session.

**Fig. 5:**
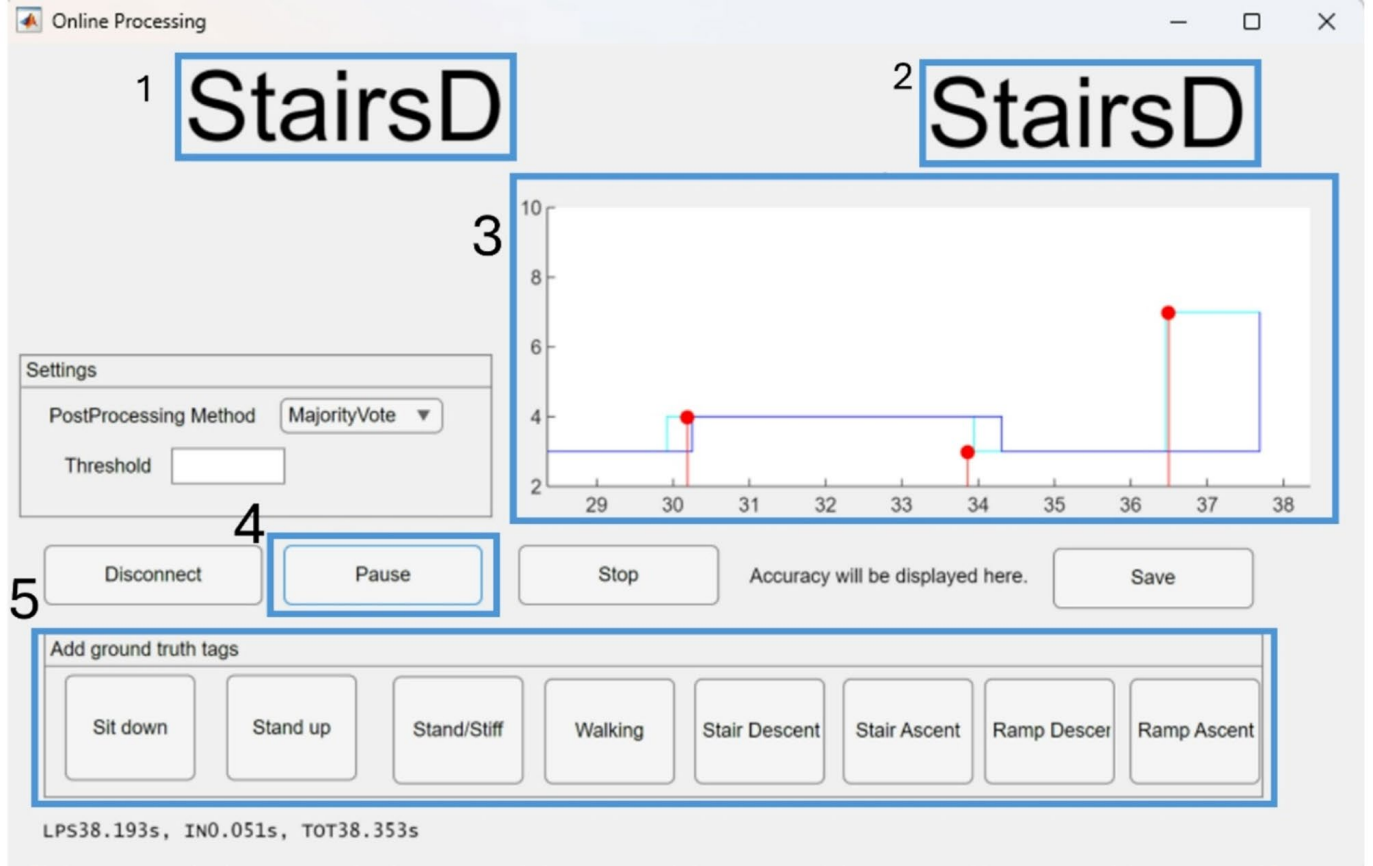
Screenshot of the real-time GUI: (1) Predicted locomotion mode (2) Ground truth entered by the conductor (3) Prediction diagrams: The red dot represents the ground truth, and the light blue indicates the predicted transition (4) Control buttons to start and pause the recording (5) Buttons to manually add the markers of the ground truth

Our data processing procedures in real-time closely mirror those employed during offline processing. This includes filtering, windowing, and labeling, all precisely synchronized with each heel contact and toe-off event. Using the classifier trained offline, we make predictions within each designated signal window. After prediction, we used a majority vote mechanism on the windows extracted in each heel contact and toe-off. This collective decision-making process determines whether a transition to a new locomotion mode is required or if the current mode should be retained (Fig. 5).

### Outcome measures

We examined two key outcome measures: prediction time of transition and locomotion detection error, and we defined two timing parameters essential to our outcome measures:

#### Critical timing

**Fig. 6:**
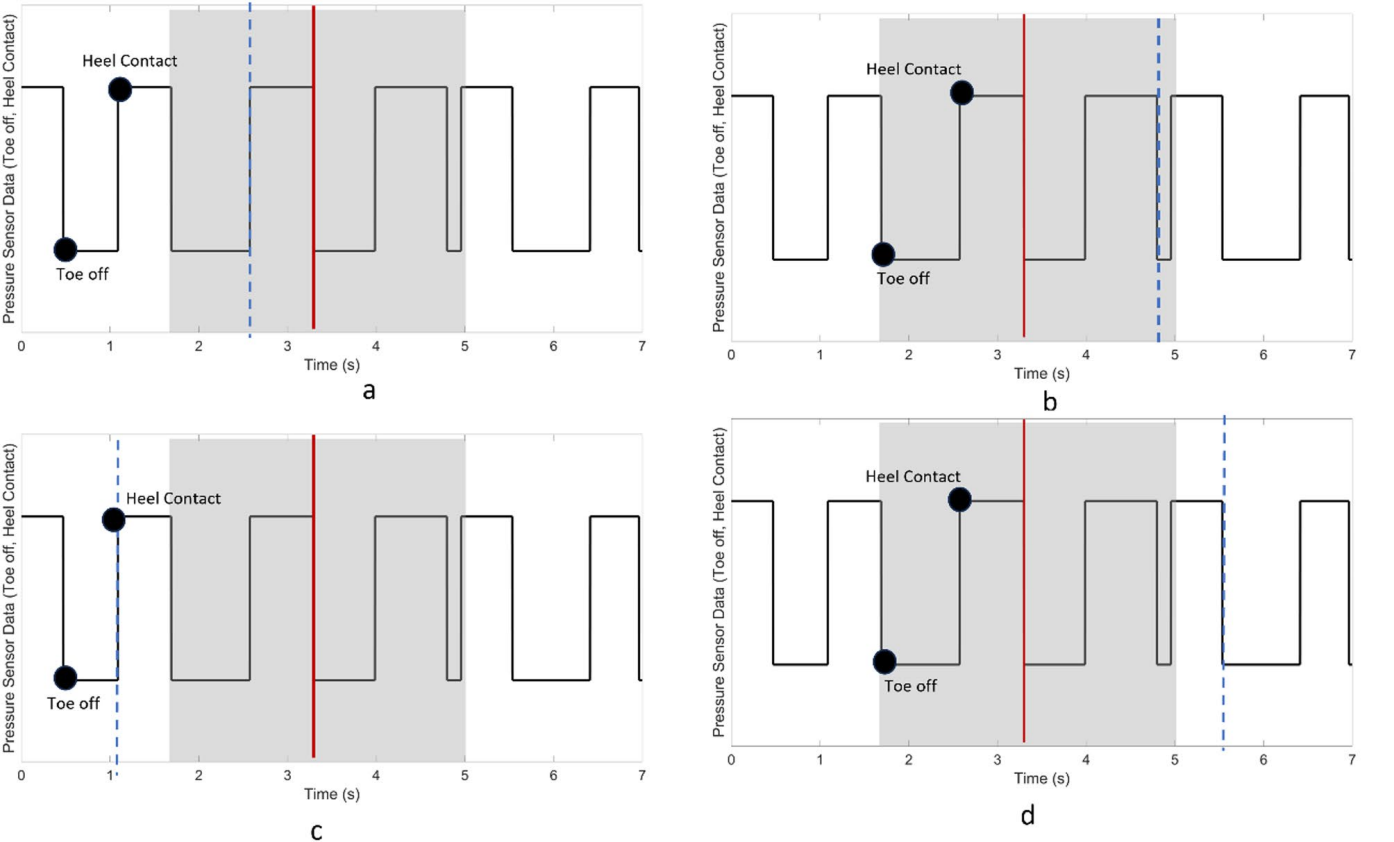
The gray area represents the transition time. The red line indicates the manually marked critical transition point, and the black signal shows the thresholded gait event state (1 = heel contact, 0 = toe-off; unitless). The blue line indicates the prediction moment. **(a)** and **(b)** Prediction occurred within the transition window and counts as correct. **(c)** Early prediction, outside the window, not counted as correct. **(d)** Late prediction, also excluded from accuracy calculation

During data recording, transition times are manually marked by the test conductor for the transition from one locomotion mode to another. These markers were then moved to the nearest toe-off or heel contact based on the transition. Critical timing signifies this moment reflecting the ideal time for prediction to occur before the leg executes the transition safely. For specific transitions like ramp descent to walking, and stair descent to walking, critical timing was defined as the beginning of the heel contact. Similarly, for transitions like stair ascent to walking, and walking to stair ascent, critical timing was defined as the initiation of the toe-off (Fig. 6).

#### Transition period

This was the time during which the algorithm can detect the transition between different locomotion modes without disrupting the participant’s movement rhythm. The period includes three distinct gait events (toe-offs and heel contacts) occurring after critical timing and two distinct gait events preceding critical timing [16] (Fig. 6). The transition between different locomotion modes is not a static process and involves more than one step. As a result, it is difficult to define a moment as the time that transition happens. We prefer to predict the transition before critical timing, but it is arguably valid to predict it within the transition period.

We defined the outcome measures as [8]:

#### Prediction time

This marks the elapsed time from the critical timing to the moment our classifier detects a transition (Fig. 6). Importantly, negative prediction time indicates the system’s response occurring before the critical event, shedding light on anticipatory predictions. The reported prediction time represents the mean average of all occurrences for each transition for each participant. A lower prediction time is deemed favorable, signifying early identification of transitions and enhanced system responsiveness.

#### Locomotion detection error (Offline)

This quantifies the percentage of windows incorrectly predicted compared to the ground truth averaged for all the extracted windows. In this step instead of using 20% of data for testing and 80% for training a 10-fold cross-validation method was used only for reporting.

#### Locomotion detection error (Online)

The classifier’s error was calculated as the percentage of incorrectly predicted windows relative to the total number of analyzed windows within the transition period. Ground truth data for error assessment was established based on the markers entered by the test conductor. To ensure that evaluation reflects functional performance, only predictions made within this defined transition window, centered around biomechanical events such as toe-off and heel contact, were considered correct. Even if a transition was correctly predicted outside the transition period, it was not counted as correct because, in a real-time prosthetic control context, early or delayed predictions could fail to trigger timely and safe mode switches. This evaluation approach ensures that the reported metric captures both the error and the practical relevance of predictions.

## Results

Table 2 presents the classification error of offline locomotion detection averaged over all the windows during the 35 trials for each participant and each transition. We also evaluated offline classification performance using three distinct sensor configurations: EMG-only, IMU-only, and EMG + IMU combined. The combined sensor configuration produced the lowest average classification error (5%), while IMU-only achieved 8%, and EMG-only performed notably lower at 25%. Although the improvement from combining EMG with IMU appears modest, it is potentially significant in the context of lower-limb prosthesis control, where even small improvements in transition detection can reduce the likelihood of instability or falls. This analysis was limited to offline data, as real-time testing was conducted only with the combined sensor configuration.

**Fig. 7:**
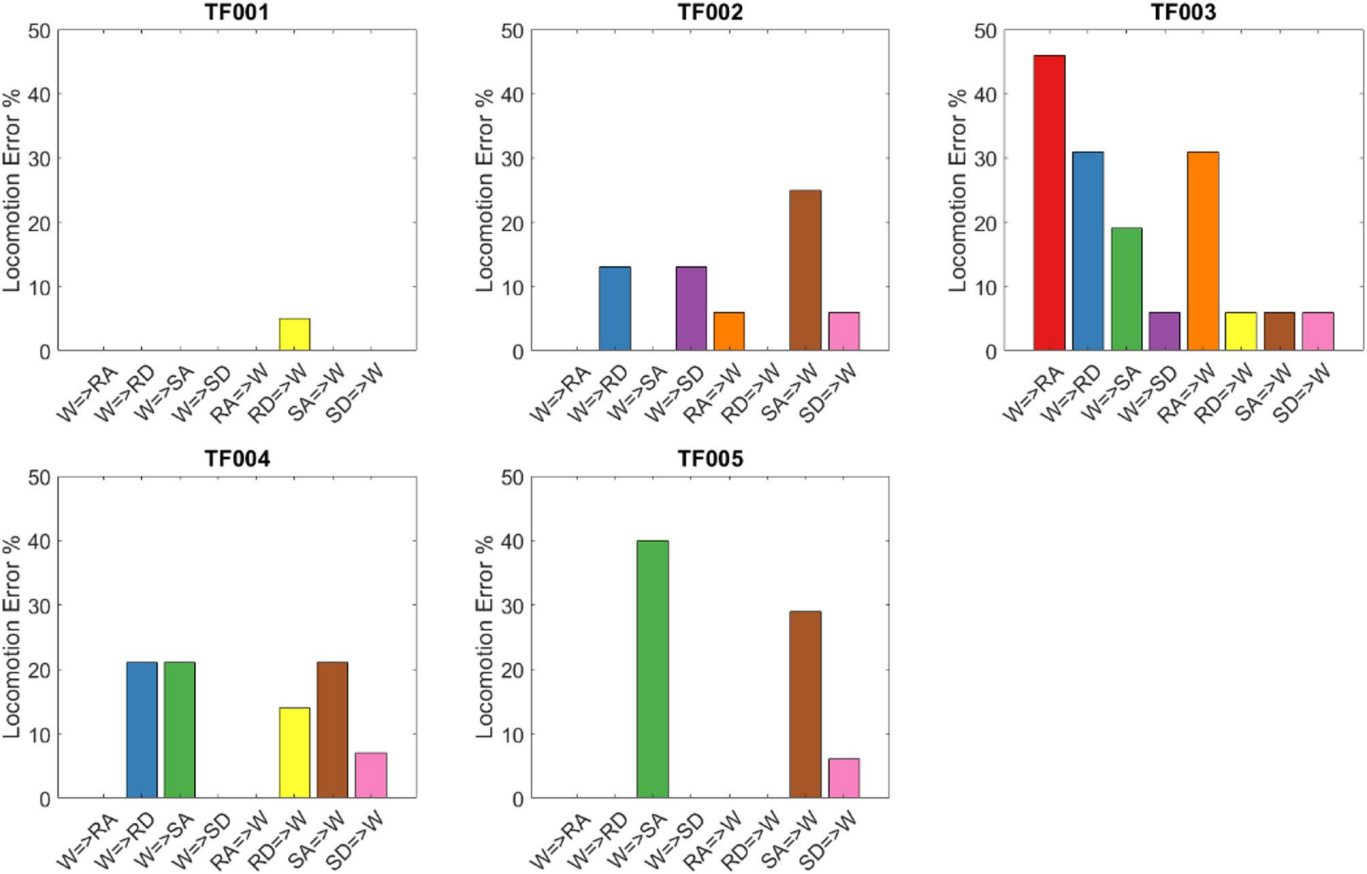
Online classification error rates for each participant during the real-time evaluation session. Each bar represents the percentage of misclassifications for a specific locomotion mode transition (e.g., level walking to ramp ascent) in the transitional period. Error rates are computed based on real-time predictions at gait events (toe-off or heel contact) and reflect the system’s performance under real-time conditions. W is walking, RA ramp ascent, RD ramp descent, SA Stair ascent, and SD stair descent

Figure 7 illustrates the real-time locomotion detection error, while the mean prediction time is presented in Table 3 for every transition type performed by each participant. Negative values in the table indicate that the predictions occurred before the critical timing of the transition. In Video 1 in complementary material, one can see the predicted tag and its delay for TF002 in real time.

**Fig. 8:**
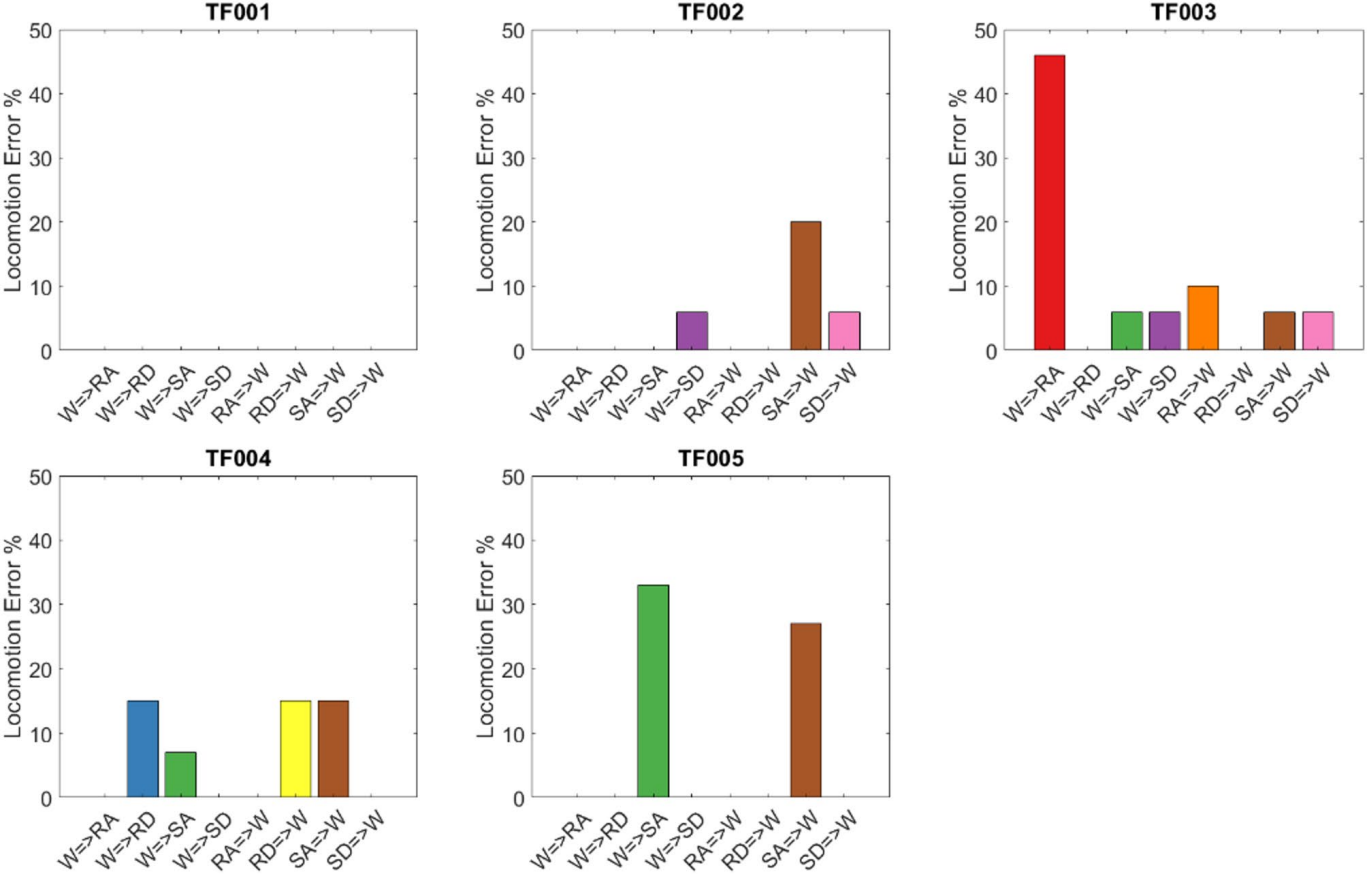
Exploratory analysis of online classification error during transitions, using a broader window of 4 toe-off/heel contacts before and 5 after the manually labeled transition point. This figure evaluates the effect of potential labeling delay or early prediction on reported classification error. The results illustrate that even when manual markers were slightly misaligned with the user’s actual intent or biomechanical transition, the classifier often predicted the correct mode within a broader functionally relevant window. W is walking, RA ramp ascent, RD ramp descent, SA Stair ascent, and SD stair descent

To assess the impact of potential labeling misalignment on reported real-time accuracy/error, we evaluated classification performance using an extended transition period including four gait events before and five after the manually marked transition point. This analysis revealed that most correct classifications occurred within this broader window, suggesting that minor labeling delays may have affected strict accuracy calculations under the original time window definition. These results are summarized in Fig. 8, which shows detection errors across all participants for each transition type (e.g., walking to ramp ascent or stair descent). The trend supports the robustness of the classifier even under moderate temporal uncertainty and highlights the importance of aligning transition labels with biomechanical events.

To summarize, the proposed real-time classification system successfully operated across all participants with transfemoral amputation and osseointegrated implants, demonstrating the feasibility of phase-dependent, mode-specific locomotion detection under real-world conditions. While overall system functionality was confirmed, classification performance varied notably between participants, with average error rates ranging from 1.2 to 23%. This variability highlights the importance of individual anatomical and physiological factors such as residual limb length, muscle availability, and EMG signal quality when designing and calibrating real-time control systems. These results underscore the potential of personalized classification approaches for improving reliability and responsiveness in clinical deployment.

**Table 2:** Offline locomotion detection error for each transition averaged over all the windows for 5 participants with transfemoral amputation

Error %	TF001	TF002	TF003	TF004	TF005
Walking to Ramp Ascent	1.7	2.35	1.62	2.78	1.08
Walking to Ramp Descent	1.12	7.43	5.17	8.19	5.75
Walking to Stair Ascent	3.36	5.51	4.29	2.94	2.56
Walking to Stair Descent	0	1.99	0	1.73	1.73
Ramp Ascent to Walking	0	0.69	4.4	0	0.22
Ramp Descent to Walking	7.14	16.91	12.5	9.82	48
Stair Ascent to Walking	4.19	12.71	12.31	3.64	13.71
Stair Descent to Walking	7.69	8.15	27.78	9.26	19.71
Mean ± SD	3.15 ± 2.8	7 ± 5.2	8.5 ± 8.4	4.8 ± 3.4	11.5 ± 15.2

**Table 3:** Prediction time (milliseconds) of different movements for 8 different transitions and 5 participants in real-time. Negative values in the table show that the predictions occurred before the critical timing of the transition. W is walking, RA ramp ascent, RD ramp descent, SA stair ascent, and SD stair descent

	W = > RA	W = > RD	W = > SA	W = > SD	RA = > W	RD = > W	SA = > W	SD = > W
TF001	452 ± 548	694 ± 462	597 ± 950	585 ± 598	966 ± 283	-34 ± 283	553 ± 784	-34 ± 497
TF002	620 ± 377	875 ± 566	1275 ± 695	757 ± 178	1212 ± 384	498 ± 706	1005 ± 818	85 ± 491
TF003	1439 ± 2500	1513 ± 828	817 ± 757	534 ± 650	1032 ± 505	73 ± 695	306 ± 702	-186 ± 333
TF004	805 ± 387	851 ± 535	1034 ± 732	912 ± 308	980 ± 446	-100 ± 0	61 ± 535	-240 ± 506
TF005	666 ± 827	773 ± 762	1637 ± 1700	1177 ± 700	896 ± 696	-100 ± 0	176 ± 823	-981 ± 458
Mean	796 ± 340	941 ± 292	1072 ± 361	793 ± 233	1017 ± 106	67 ± 224	420 ± 335	-271 ± 373

## Discussion

In our study, we explored the feasibility of real-time locomotion detection in short residual limbs with skeletal attachment of prosthetic legs, and utilizing the open-source platform LocoD. Recent research in the realm of lower limb control has unveiled promising outcomes in the domain of real-time locomotion [4, 5, 8]. Against this backdrop, our main objective was to study how EMG integration impacts real-time detection of locomotion modes in participants with osseointegrated implants. We measured the prediction error during transitions and the associated delay concerning critical timing, offering insights into the usability of this setup.

Starting with offline data (Table 2), our investigation mirrored previous literature, revealing negligible errors [9]. A closer look at individual participant results hinted at intriguing connections. For instance, Participant 1, who exhibited outstanding real-time performance, also showcased minimal error in offline scenarios, highlighting a noteworthy correlation. However, it is not possible to make a complete conclusion based on the offline results as they are not correlated for all the participants and all the movements. There is a known difference between offline and real-time decoding in upper limb prosthesis [36, 37]. In lower limbs, the disparities between offline and real-time performance for each specific transition could also be caused by the variability in transition markers’ timing and the nature of movements, accentuating the complexity of our study and the different methods of classification between offline and real-time scenarios. Several previous studies have evaluated both offline and real-time locomotion mode classification systems, consistently reporting lower error in offline analyses. For instance, Zhang et al. observed that static state classification error increased from 2% offline to 5% in online use [10]. Similarly, Hernandez et al. achieved 3.7% real-time error in a mobile computing-based interface, with offline error of 1.2% [38]. Hargrove et al. further illustrated this pattern in a randomized clinical trial: the offline error using EMG and dynamic Bayesian networks was 2.9%, but in real-time, these configurations showed higher absolute errors 7.9% [39]. While such results confirm the utility of EMG and temporal modeling in decreasing error, they also emphasize that offline performance gains do not necessarily reflect real-time usability. Offline evaluations are conducted under idealized conditions whereas real-time classification must contend with variable signal quality, decision deadlines, and practical deployment constraints.

During the real-time experiment, we uncovered a range of error rates during transitions. Participant performance, illustrated in Fig. 7, showcased Participant 1 with near-zero error across all transitions—a highly encouraging outcome. Participants 2 and 5 demonstrated commendable performance, while Participants 3 and 4 showed less reliable accuracy, underlining the participant-dependent nature. Importantly, our results suggest that EMG holds promise in detecting all movements for some participants, while for others, it may be effective only in detecting a subset of transitions, such as stair descent to walking—a challenging task for commercial devices, often necessitating unnatural hip movements from users [40]. Additionally, the use of a two-phase classifier in our study, restricted to predictions at heel contact and toe-off also contributed to the observed prediction delays. This choice was a consequence of our sensor configuration, which relied on a uniaxial ground reaction force sensor that enabled robust detection of only these two gait events. In contrast, studies such as [9] utilized four-phase models, allowing more frequent decision points per stride. Our reduced temporal resolution, while ensuring reliability and simplicity in event detection, may delay transition recognition by up to half a stride, particularly in slower gait cycles. We consider this a trade-off that reflects the practical constraints of our real-time setup, and an important direction for future improvement.

It is important to acknowledge that the metrics employed in this study may not fully capture classifier performance, partly due to potential human error and the dynamic nature of locomotion transitions [8]. Certain movements, such as transitions between ramp ascent and level walking, are particularly challenging to detect accurately at entry, especially given their biomechanical similarity. In fact, some commercial prosthetic systems do not distinguish ramp ascent from level walking and lack a dedicated mode for ramp navigation [27]. A significant source of uncertainty in our results stems from the manual entry of transition markers, which, despite visual monitoring, inevitably introduced reaction time delays and potential misalignment with actual biomechanical events. To mitigate these effects, we applied an automatic synchronization process that adjusted manually entered markers to the next relevant gait event (either heel contact or toe-off). However, recognizing that minor discrepancies could still persist, we further extended the transition period to include four gait events before and five after the manually entered transition point. As shown in Fig. 8, this expanded window led to a reduction in error rates across all participants and transitions, suggesting that some misclassifications were attributable to marker misalignments rather than true classifier failures. Beyond marker misalignment, anatomical differences among participants also likely influenced classification performance. Users of osseointegrated prostheses often have different residual limb characteristics compared to socket users, including shorter stumps or soft tissue challenges that preclude traditional socket fitting. While osseointegration resolves socket-related issues such as relative electrode motion and signal distortion, participants with shorter residual limbs may have fewer available muscles for recording, complicating EMG-based control. Muscle activation patterns and available muscle mass are critical factors influencing classification accuracy.

An important limitation of this study is the use of passive prosthetic legs during both training and online evaluation. Although this approach enabled safe and controlled experimentation, it does not replicate the dynamic interactions between user and device that would occur with powered prostheses. Powered knees and legs introduce additional torque and damping, which can alter surface EMG patterns and impact classifier behavior. The absence of real-time device feedback in passive systems may limit users’ ability to adapt their activation patterns or recognize and correct misclassifications. In contrast, active prostheses inherently provide behavioral feedback through changes in joint motion or resistance, which could support more effective training and promote consistent user-classifier interaction. Future work must therefore include active prosthetic legs to validate classifier performance under realistic biomechanical conditions, and explore the benefits of integrated feedback during both training and daily use. Additionally, the classifier in this study did not include a resting or standing mode, which is a common state in daily life. The exclusion of this mode reduces the ecological validity of the system and may overlook important transition scenarios, such as initiating gait from a static position. Future studies should therefore include a standing mode to better reflect real-world use and to improve the continuity of mode detection across daily movement tasks.

We also acknowledge the inherent differences between surface and implanted EMG signals in terms of signal quality, stability, and selectivity. While surface EMG may not offer the long-term viability that implanted systems promise, it serves as a critical intermediate step enabling us to evaluate classification strategies and system feasibility before advancing to more invasive interfaces. We also recognize the trade-offs involved: although osseointegration eliminates the need for a socket, skin-mounted electrodes may reintroduce challenges related to comfort and electrode stability. Nonetheless, we regard this approach as translationally informative, not as a replacement for implanted solutions but as a stepping stone towards them.

These findings also highlight several future directions. One promising avenue is improving EMG signal quality. Surface EMG is subject to deterioration during dynamic movement due to sweat, skin impedance changes, and electrode motion. Direct implantation of electrodes into muscles could substantially improve signal reliability, although this approach carries surgical risks and is invasive. Moreover, the importance of user training cannot be overstated. Training users with feedback during walking practice may help them develop more consistent activation patterns and improve classifier reliability. In this study, users did not receive feedback regarding decoding during training or real-time testing. Classifier predictions were displayed on a monitor visible only to the test conductor and did not influence prosthesis behavior, as participants used passive limbs. This absence of feedback may have limited users’ ability to adapt or refine their motor strategies. Future studies should incorporate visual or auditory feedback systems to support training. For example, showing real-time classifier predictions or providing haptic cues during mode transitions could enhance user-system interaction. When controllable prostheses are used, users will also receive implicit feedback through changes in device behavior during movement, further reinforcing learning and potentially improving control performance.

Future research should aim to address the limitations identified in this study. Recruitment of a larger and more diverse participant pool will be critical to generalize findings. Incorporating active prosthetic components during training and evaluation, developing training protocols with real-time feedback, implementing reset mechanisms to recover from misclassifications, exploring implanted electrode solutions, and automating locomotion mode labeling are all necessary steps toward translating EMG-driven control into real-world clinical applications.

## Conclusion

In conclusion, our study provides insights into the potential application of myoelectric signals for controlling lower limb prosthetic devices. Leveraging open-source software and data collected from individuals with transfemoral amputation and osseointegration, we successfully demonstrated the feasibility of real-time locomotion prediction using both EMG signals and IMUs. Our outcomes, in harmony with existing literature, underscore the advantages of incorporating bioelectric signals.

It’s crucial to acknowledge that the current technology is not yet ready for widespread deployment in take-home devices. Challenges with delays in finding the transitions and issues related to the quality of EMG signals during dynamic movements highlight this technology’s limitations. Participant-dependent outcomes revealed nuances influenced by residual limb length and muscle availability, underscoring the complexity of the problem.

Looking forward, we recommend future research to refine these shortcomings. Exploring active prosthetics, advanced control algorithms, and investigating the benefits of using implanted electrodes for recording EMG signals offer promising avenues. These recommendations aim to address current limitations and propel the development of prosthetic devices toward heightened functionality, ultimately enhancing the quality of life for individuals with lower limb amputation.

## Electronic supplementary material

Below is the link to the electronic supplementary material.

Supplementary Material 1

## Data Availability

The datasets gathered and analyzed during the current study are available in the Zenodo repository at https://zenodo.org/records/14652460. The software developed for this study is publicly accessible on GitHub at https://github.com/biopatrec/LocoD.
